# The Use of Quercetin to Improve the Antioxidant and Regenerative Properties of Frozen or Cryopreserved Human Amniotic Membrane

**DOI:** 10.3390/antiox11071250

**Published:** 2022-06-25

**Authors:** Valeria Purpura, Serena Benedetti, Elena Bondioli, Francesca Scarpellini, Agnese Giacometti, Maria Cristina Albertini, Davide Melandri

**Affiliations:** 1 O.U. Burn Center and Emilia Romagna Regional Skin Bank, “M. Bufalini” Hospital, AUSL Romagna, 47521 Cesena, FC, Italy; elena.bondioli@auslromagna.it (E.B.); davide.melandri@auslromagna.it (D.M.); 2 Department of Biomolecular Sciences, University of Urbino Carlo Bo, 61029 Urbino, PU, Italy; serena.benedetti@uniurb.it (S.B.); maria.albertini@uniurb.it (M.C.A.); 3 O.U. Pathological Anatomy, “M. Bufalini” Hospital, AUSL Romagna, 47521 Cesena, FC, Italy; francesca.scarpellini@auslromagna.it (F.S.); agnese.giacometti@auslromagna.it (A.G.)

**Keywords:** burns, non-healing wounds, cryopreservation, freezing, wound treatment, regenerative properties, growth factors, lipid peroxidation, natural antioxidant compound

## Abstract

The biological properties of the human amniotic membrane (HAM) and its characteristic ability to be a reservoir of growth factors promoting wound healing make it an ideal biological dressing for the treatment of different clinical conditions, such as burns and non-healing wounds. However, the application of a preservation method on the HAM is required during banking to maintain biological tissue properties and to ensure the release overtime of protein content for its final clinical effectiveness after application on the wound bed. Although cryopreservation and freezing are methods widely used to maintain tissue properties, reactive oxygen species (ROS) are produced within tissue cellular components during their switching from frozen to thawed state. Consequently, these methods can lead to oxidative stress-induced cell injury, affecting tissue regenerative properties and its final clinical effectiveness. Taking advantage of the antioxidant activity of the natural compound quercetin, we used it to improve the antioxidant and regenerative properties of frozen or cryopreserved HAM tissues. In particular, we evaluated the oxidative damage (lipid peroxidation, malondialdehyde) as well as the regenerative/biological properties (bFGF growth factor release, wound healing closure, structure, and viability) of HAM tissue after its application. We identified the effectiveness of quercetin on both preservation methods to reduce oxidative damage, as well as its ability to enhance regenerative properties, while maintaining the unaltered structure and viability of HAM tissue. The use of quercetin described in this study appears able to counteract the side effects of cryopreservation and freezing methods related to oxidative stress, enhancing the regenerative properties of HAM. However, further investigations will need to be performed, starting from these promising results, to identify its beneficial effect when applied on burns or non-healing wounds.

## 1. Introduction

The human amniotic membrane (HAM) is a thin tissue derived from the placenta, structurally composed of three different layers that cover and protect the embryo during the uterine life [1]. The absence of ethical problems for its clinical use and its beneficial properties to the wound bed make it very attractive for different clinical applications [1,2], and an ideal candidate as a biological dressing for the treatment of burns [3,4,5] and non-healing wounds [6,7,8]. To maintain the structural and biological properties of the HAM until its clinical use, a preservation method is required to be applied by tissue banks. Among the different types of preservation methods used for banking, cryopreservation, as well as freezing, are frequently chosen for their ability to better preserve biological characteristics such as structure, cell viability, and growth factors content compared to other methods [9]. Although these storage methods are similar, they differ in the temperature achieved from tissue, respectively, −150 °C (Cryopreservation protocol 1; CP-1) and −80 °C (Freezing protocol 2; FP-2). Taking into account the relationship between HAM viability and the release of proteins involved in homeostasis and wound healing [10], cryopreservation and freezing appear to be methods suitable for the preservation of HAM, to promote wound healing after its thawing for clinical applications [11,12]. However, the freezing-thawing of the tissue, required in both preservation methods, can induce cellular stress due to reactive oxygen species (ROS) production, leading, in turn, to cell damage such as lipid peroxidation and rupture of the mitochondrial, as well as the plasma membrane [13]. Thus, strategies able to improve the cellular well-being of cryopreserved or frozen biological samples were previously developed for their final clinical effectiveness [14,15]. For the same purpose, we decided to develop and compare two preservation protocols aimed at preventing ROS production within HAM tissue. In particular, we take advantage of the reported ability of quercetin as a natural antioxidant compound to prevent ROS production when applied to human samples of clinical interest [16,17]. In this way, we propose to enhance the biological properties of the HAM, and, in turn, improve its clinical effectiveness for wound treatment.

## 2. Materials and Methods

### 2.1. Procurement and Processing of HAM

The HAM was separated from the placenta of the donor after an elective cesarean operation following the National Rules on harvesting, processing, and distributing tissues for transplantation [18]. HAM tissue was then rinsed with 0.9% NaCl solution and stored in a transport solution (at 2–10 °C) to the tissue bank (<12 h), where it was aseptically processed or immediately used for analysis. In detail, HAM was cut in grafts of 4 × 4 cm, placed in T25 culture flasks, and immersed in 6 mL of RPMI 1640 medium plus antibiotics and 10% dimethyl sulfoxide (DMSO Alchimia Srl, Ponte San Nicolò, Italy) for 15 min in the presence or not of quercetin (50 µM, Sigma-Aldrich, Milan, Italy). Then, HAM grafts were packaged into sterile bags and alternatively cryo-frozen with a programmed gradual drop in the temperature to −150 °C and then stored at −80 °C (cryopreservation protocol 1, CP-1) or frozen and stored immediately at −80 °C (freezing protocol 2, FP-2). The gradual temperature drop until −150 °C started slowly with a speed of 1 °C/minute for 15 min. Then, a drastic drop of the temperature to −20 °C in 5 min was applied and followed by another final slow drop of 1 °C/minute for 45 min and 5 °C/minute for 20 min. Tissue sterility was routinely guaranteed by Tissue Bank’s internal protocols, which consist of microbiological analyses on HAM before and after its processing/storage. Briefly, small pieces of HAM were incubated on plates selective for the growth of bacteria (COS Columbia agar + 5% sheep blood, BioMerieux, Marcy-l’Etoile, France) or fungi (Sabouraud agar, BioMerieux, Marcy-l’Etoile, France) for 3 or 14 days, respectively. Thus, the sterile conditions of our samples were maintained during all procedures, excluding the possibility that our results could be influenced by the presence of contaminants.

The human amniotic membrane was collected and supplied by Emilia Romagna Regional Skin Bank with the authorization of the Regional and National Transplant Center. The activities described in this study are exclusively aimed at improving the quality and better characterization of the tissue intended for human transplantation and do not require approval by an ethics committee.

### 2.2. Collection of HAM-Derived Supernatant

HAM grafts of 4 × 4 cm stored using CP-1 or FP-2 in the presence or not of quercetin were thawed in saline solution, dried using sterile gauze, placed in T25 culture flasks, and then incubated in 6 mL of RPMI 1640 serum-free culture medium plus antibiotics for 40 min (T0) or 3 days at 37 °C and 5% CO_2_ (T3). Then, the supernatant was collected and used for different analyses (MDA HPLC quantification, bFGF ELISA test, wound healing closure).

#### 2.2.1. Malondialdehyde Determination

Malondialdehyde (MDA) is a marker of oxidative stress and, in particular, lipid peroxidation. It is a reactive aldehyde that forms adducts with proteins and DNA, leading to alterations in cellular function [19]. MDA levels after HAM thawing were evaluated in HAM-derived supernatants by RP-HPLC as previously described [20]. Briefly, sample derivatization was carried out by adding 50 µL 0.05% butylated hydroxytoluene (BHT), 400 µL 0.44 M H_3_PO_4,_ and 100 µL 42 mM thiobarbituric acid (TBA) to 50 µL supernatant. Tubes were heated for 1 h at 100 °C and 250 µL of butanol was then added to extract the MDA-TBA complex. After centrifugation at 10,000× *g*, the butanol layer was collected and immediately used for MDA analysis into an HPLC system from Jasco Corporation (Tokyo, Japan). The assay was performed using an Alltima C18 column (4.6 × 250 mm, 5 µm, Alltech, Milan, Italy); the eluent phase consisted of methanol/50 mM KH_2_PO_4_, pH 6.8 (40:60, *v/v*), with a flow rate of 0.8 mL/min. The fluorescence detector was set with an excitation/emission wavelength of 515/553 nm. Data were expressed as mean ± SD and paired *t*-test was applied to evaluate statistical differences. Significance was set at *p* < 0.05.

#### 2.2.2. bFGF ELISA Test

To evaluate the release of basic fibroblast growth factor (bFGF) from HAM, we performed an ELISA test using the Quantikine^®^ ELISA human FGF basic immunoassay kit (R&D Systems Inc., Minneapolis, MN, USA). The measured optical density was proportional to the protein content (pg/mL) of the HAM-derived supernatants. Results were expressed as fold change bFGF pg/mL related to control. The relative bFGF amount/mL mean values +/− standard deviation were reported in the corresponding figure. The value of *p* was calculated using Student’s *t*-test and the significance level was defined as *p* < 0.05.

#### 2.2.3. Wound Healing Closure

Primary human fibroblasts were seeded in 24 multi-well plates and cultured until the formation of a cell monolayer. Then, a mechanical scratch wound was performed on confluent cells, horizontal along the well, using the tip of a sterile syringe. Human fibroblasts were incubated at 37 °C and 5% CO_2_ for 48 h in the presence of HAM-derived supernatants obtained as described above. The experimental protocol has been previously validated by verifying that fibroblasts could grow similarly in the HAM supernatant (collected after 3 days) and serum-free medium without cell death. Supernatants from fresh not preserved HAM tissues were used as controls. Starting from the time immediately after scratching (0 h), images of the same wound area were acquired after 48 h using a phase-contrast microscope (Leica Microsystems). The total migration area during wound closure was identified using ImageJ software (version 1.45, National Institutes of Health, Bethesda, MD, USA). The wound closure was expressed as a percentage after the application of the formula described below:

[(Wound area 0 h − Wound area 48 h)/Wound area 0 h] × 100.(1)

The results are expressed as mean values ± standard deviation (SD). The value of *p* was calculated using Student’s *t*-test and significance levels are defined as *p* < 0.05.

### 2.3. Histological Analysis

Histological analysis was performed on the HAM graft, stored with CP-1 or FP-2 in the presence or not of quercetin after its processing/storage in order to compare the maintenance of structural and cellular integrity after the treatment with quercetin. In all cases, small pieces of HAM were fixed with a 10% formalin solution and paraffin embedded. After processing, histological sections of 5-µm in thickness were stained with hematoxylin and eosin. Histological analysis on HAM untreated and not preserved was used as a control.

### 2.4. Cell Viability Test (MTT)

The MTT test was performed on HAM grafts stored with CP-1 or FP-2 in the presence or not of quercetin in order to evaluate the maintenance of cell viability. In all cases, six uniform HAM samples were obtained using a biopsy punch 0.5 µm in diameter. Tissue specimens were weighed and incubated with MTT solution (Roche Diagnostic GmbH, Mannheim, Germany) at 0.5 mg/mL for 3 h at 37 °C. Then, all samples were placed in DMSO (Alchimia Srl, Italy) for 10 min and the colored solution obtained, which is directly proportional to cell viability, was read using a spectrophotometer set to 570 nm. Quantitative analysis was performed to assess the viability rate of all samples, calculated as the ratio between the optical density (OD) at 570 nm and HAM weight in grams (g). The results are expressed as mean values of OD/g ± standard deviation (SD). The value of *p* was calculated using the Student’s *t*-test and the significance level is defined as *p* < 0.05.

## 3. Results

### 3.1. The Pre-Storage Treatment of HAM with Quercetin Reduces Oxidative Stress after Its Thawing

**Figure 1:**
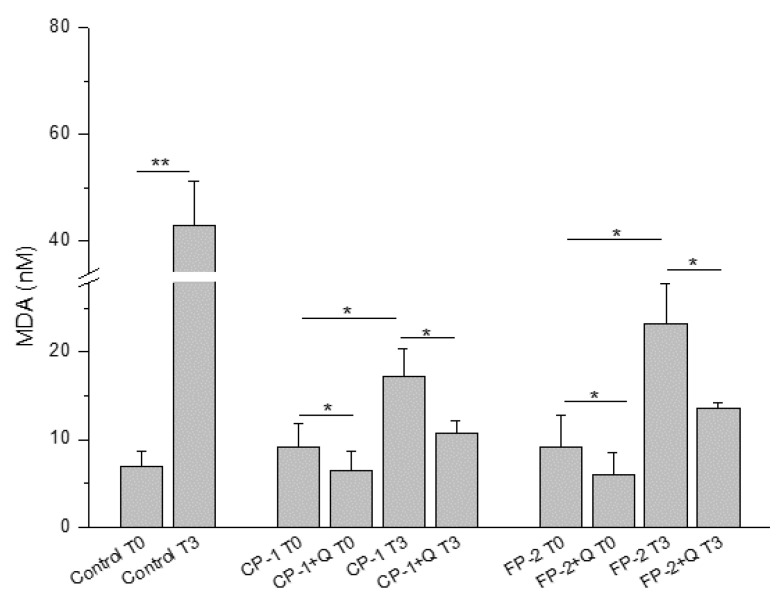
MDA levels in HAM-derived supernatants obtained immediately after thawing (T0) and after 3 days of HAM culture (T3). Control: supernatants from fresh not preserved HAM; CP-1: cryopreservation protocol 1; FP-2: freezing protocol 2; Q: quercetin. Data are represented as mean ± SD of 3 independent experiments. * *p* < 0.05 and ** *p* < 0.01 between the indicated bars.

Increased oxidative stress after the application of cryopreservation methods was previously identified for different human biological samples [21,22]. However, no data were suitable in the literature regarding oxidative stress induced by cryopreservation or freezing on HAM tissue; consequently, we decided to evaluate it on our samples. As shown in Figure 1, the use of quercetin was able to significantly reduce the lipid peroxidation induced by both CP-1 and FP-2 methods of preservation (−31% and −35%, respectively), suggesting its antioxidant effect on the cellular component of HAM tissue. The antioxidant protection of quercetin was evidenced not only immediately after thawing (T0), but also after 3 days of HAM culture in RPMI 1640 medium (T3), when a significant MDA increase was observed in comparison to T0. Indeed, 3-day supernatants derived from HAM tissues treated with quercetin before cryopreservation/freezing presented a significant reduction in MDA levels compared to samples from HAM tissue not treated with quercetin before storage (−37% and −41%, respectively).

### 3.2. The Antioxidant Effect of Quercetin Improves the Biological Properties of HAM Tissue after Its Thawing

**Figure 2:**
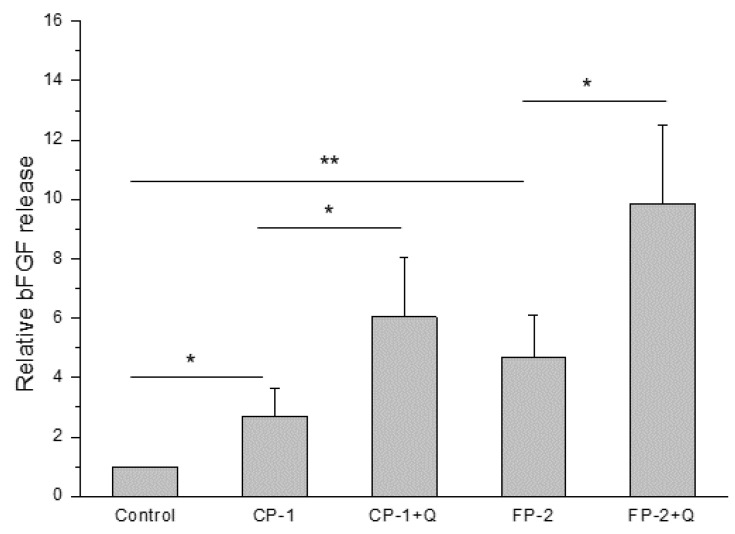
Release of basic fibroblast growth factor (bFGF) after 40 min from fresh HAM samples (Control) and thawed HAM, previously preserved with CP-1 or FP-2 in the presence (CP-1 + Q and FP-2 + Q) or not (CP-1 and FP-2) of quercetin. Data are expressed as fold change bFGF pg/mL related to control and are represented as mean ± SD of 3 independent experiments. * *p* < 0.05 and ** *p* < 0.01 between the indicated bars.

To investigate if the quercetin treatment could improve the biological properties (growth factor production and release) of stored HAM, we also evaluated tissue bioactivity. In fact, it plays an important role in stimulating wound healing when stored HAM is thawed and applied for clinical purposes, so preserved methods are preferred if its properties are maintained after tissue storage. In particular, we performed an ELISA test to evaluate the ability of HAM to release the basic fibroblast growth factor (bFGF), since it is a growth factor widely involved in the healing process of wounds [23,24,25]. As shown in Figure 2, the addition of quercetin to the storage methods CP-1 or FP-2 significantly increased the release of bFGF in the supernatant derived from post-thawed HAM compared to stored and not treated tissue. Thus, the antioxidant effect of quercetin on HAM cellular components was able to improve the release of growth factors involved in tissue regeneration, maintaining, in turn, tissue bioactivity after thawing.

### 3.3. The Use of Quercetin Stimulates Wound Healing Closure

**Figure 3:**
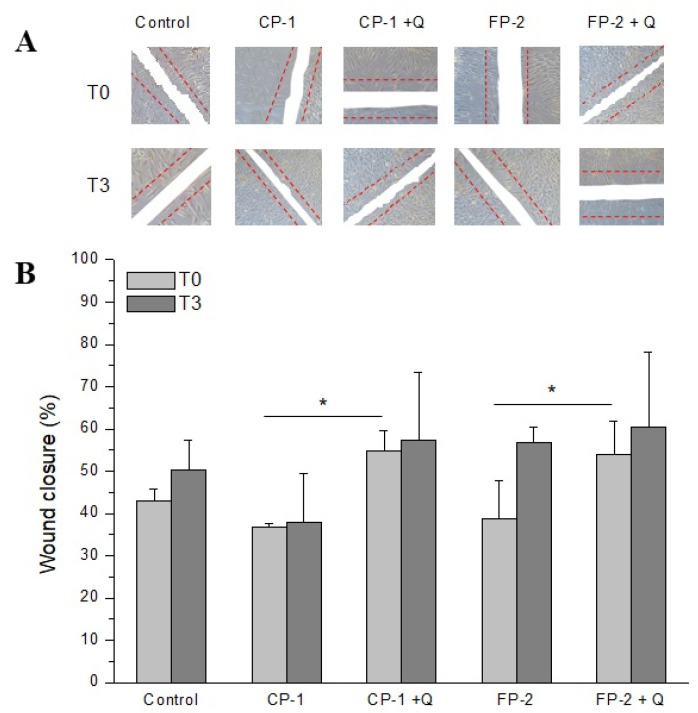
Percentage of fibroblast wound closure after 48 h treatment with supernatants derived from fresh not preserved HAM tissues (Control) or thawed HAM samples preserved with CP-1 or FP-2 in the presence (CP-1 + Q and FP-2 + Q) or not (CP-1 and FP-2) of quercetin. T0: supernatants collected after 40 min of HAM culture; T3: supernatants collected after 3 days of HAM culture. (**A**). Representative images used to evaluate the percentage of wound closure illustrated in (**B**). In the same figures, the initial wound (0 h) has been evidenced with dotted red lines while the wound after 48 h is indicated as a white area. The percentages of wound closure are represented as mean ± SD of 3 independent experiments. * *p* < 0.05 between the indicated bars.

Taking into account our previous results on quercetin-induced bFGF release, we evaluated if this effect could stimulate wound healing. To this end, we performed an in vitro wound-healing assay as described in the materials and methods. Our results showed that human fibroblasts treated for 48 h with supernatants derived from thawed HAM samples stored with CP-1 or FP-2 were able to proliferate and cover the empty area more quickly when quercetin was added (Figure 3B, T0). Although the quercetin-induced wound closure was not evident with supernatants from HAM samples cultured for 3 days (Figure 3B, T3), the results were comparable to the other samples. These findings could be related to the serum-free culture condition of HAM tissue before collection of the supernatant after 3 days. In fact, we decided to test bFGF release in the same samples used for wound closure analysis, so that the use of fetal bovine serum was not permitted. Thus, it is possible that cells of HAM tissue produce a high amount of ROS when this condition is prolonged over time (3 days), as identified in our first experiment when quercetin could not completely counteract this effect.

### 3.4. The Use of Quercetin Maintains the Structural Properties and Cell Viability of HAM Tissue after Its Thawing

**Figure 4:**
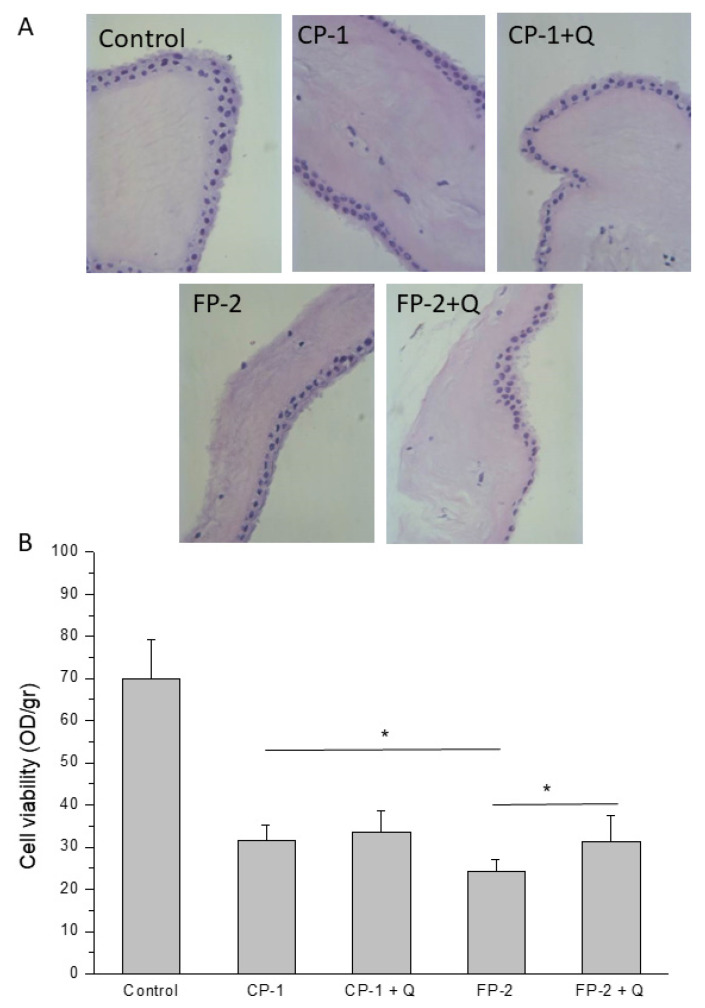
(**A**) Histological evaluation immediately after thawing of HAM preserved with cryopreservation protocol 1 (CP-1) (upper, central panel) or with CP-1 in the presence of quercetin (CP-1 + Q, upper right panel) and HAM preserved with freezing protocol 2 (FP-2) (lower, left panel) or with FP-2 in the presence of quercetin (FP-2 + Q, lower right panel). Untreated and not preserved HAM was used as control (upper, left panel). (**B**) MTT viability test on fresh not preserved HAM tissues (Control) and thawed HAM samples preserved with CP-1 or FP-2 in the presence (CP-1 + Q and FP-2 + Q) or not (CP-1 and FP-2) of quercetin. Data are represented as the mean of OD/gr ratio ± SD of 3 independent experiments. * *p* < 0.05 between the indicated bars.

Taking into account the previous results, we decided to evaluate the effect of quercetin on both cellular components and structural properties of HAM. To this end, HAM grafts were stored using CP-1 or FP-2 in the presence or not of quercetin, and then we tested their structural integrity and cellular viability immediately after thawing. Our results showed that HAM treated with quercetin and preserved using CP-1 or FP-2 presented structural integrity comparable to the corresponding stored, untreated tissue (Figure 4A).

HAM graft preserved with CP-1 showed a number of viable cells after thawing slightly, but significantly higher than the same tissue stored with FP-2. The use of quercetin in addition to CP-1 maintained the cell viability of HAM tissue comparable to the corresponding stored untreated tissue (Figure 4B). On the other hand, cell viability resulted slightly but significantly increased after the addition of quercetin to FP-2 (Figure 4B).

## 4. Discussion

The advantageous biological properties of the human amniotic membrane are historically known and its use as an alternative to other biological skin substitutes [26] was already suggested in the early 1980s for the treatment of chronic skin ulcers in combination with autograft skin [27]. To date, the use of HAM for the treatment of different clinical conditions such as burns or skin wounds has been strongly supported by different authors [3,4,5,6,7,8], making it a powerful biological medication. However, its use over time requires the application of a preservation method, raising the problem of how to ensure its effective biological properties remain unaltered as much as possible.

Cryopreservation, as well as freezing, is frequently used as a preservation method by tissue banks to maintain different HAM biological properties such as structure, cell viability, as well as growth factor and protein content [9]. The number of viable cells is always reduced compared to fresh HAM but it can be differently maintained based on the specific protocol applied [28,29,30,31,32,33]. However, in both methods of preservation, the required passage of tissue from −150 °C or −80 °C to room temperature before its clinical use leads to oxidative stress within cells [13]. To take advantage of HAM properties maintained after cryopreservation or freezing, reducing the undesirable effect of increased ROS production after tissue thawing, we decided to perform a treatment with quercetin on our HAM samples before their storage, similarly to other studies in which antioxidants were used to improve well-being after tissue thawing [14,15,34]. The selection of quercetin as an antioxidant for our experiments was carried out based on evidence of its effectiveness at low concentration (50 µM) for the cryopreservation of other biological samples, such as human spermatozoa [35]. In fact, it was important to evaluate compounds supported by the literature and used at very low concentrations to avoid potential toxic effects when applied to tissues used for clinical purposes.

The significant decrease in lipid peroxidation identified in all HAM samples pre-treated with quercetin was the first important evidence to proceed with the next investigation, which aimed to evaluate the maintenance of tissue properties. In particular, quercetin was able to reduce oxidative stress not only immediately but also after 3 days of HAM culture, demonstrating a lasting effect over time (Figure 1). Taking into account the potential clinical use of these new protocols, we first evaluated regenerative tissue properties, identifying a promoting effect on bFGF release induced by quercetin treatment, probably related to its ability to maintain cell well-being in HAM tissue (Figure 2). However, we noticed that it was not directly related to tissue viability. In fact, the storage of HAM tissue, independently of the method used, increased the release of bFGF compared to not preserved control, in which high cell viability is naturally owned (Figure 4B). Similarly, the bFGF release appears to be higher in HAM after the application of the FP-2 method instead of CP-1, in which we identified a slightly but significantly higher number of viable cells in our experiments (Figure 4B). We can hypothesize that the higher release of bFGF identified after tissue storage and, in particular, in FP-2-stored HAM, could be partially related to the less structural integrity of these samples, with a consequent easier release of growth factors embedded in the extracellular matrix. In any case, we identified a promoting effect of quercetin on bFGF release and, in turn, on HAM regenerative properties, which was confirmed by the increased wound closure ability of quercetin-treated HAM samples (Figure 3). Even in this case, we noticed that similarly to the supernatant of HAM stored with CP-1 or FP-2, the supernatant derived from not preserved HAM tissue shows a comparable wound healing promotion (Figure 3), further suggesting just a partial role of cell viability on HAM effectiveness. In addition to its regenerative properties, the quercetin treatment does not affect the structural properties of HAM samples and it was able to maintain levels of viable cells comparable to, or slightly higher than, CP-1- or FP-2-stored samples (Figure 4), further demonstrating its additional value to the preservation of tissue properties. Although cell viability after the application of CP-1 or FP-2 appears reduced compared to not stored HAM, as expected [30], satisfactory levels of viable cells were evident after tissue thawing. Certainly, FP-2 was able to induce a higher release of bFGF growth factor, mainly involved in tissue regeneration. However, to evaluate the final effect of preservation methods on regenerative stimulus, different parameters and the way they are influenced by storage conditions have to be taken into account. For the purpose of our study, only non-frozen “fresh” HAM without quercetin is used as a control. Non-frozen “fresh” HAM with quercetin control was missing, therefore it is not clear whether quercetin affects only freezing/thawing of the sample or if it actually has a direct effect in downstream assays as well.

## 5. Conclusions

In clinical practice, the use of HAM tissues is certainly of fundamental value for wound treatments and its storage is essential to afford the high amount of patient requests. For this reason, besides HAM freezing procedures, the addition of an antioxidant molecule may improve the storage quality by maintaining the biological properties of the HAM.

Herein, we can state that the use of quercetin seems able to counteract the side effects of cryopreservation and freezing related to peroxidation and induction of oxidative stress inside cells, leading, in turn, to a higher tissue well-being and an enhancement of the regenerative properties of HAM required for wound healing. However, the results obtained in this study can be considered a starting point for further investigations on the employment of quercetin during other experimental conditions, before or after the freezing steps, to better understand how its antioxidant activity may be exploited to improve HAM preservation until clinical application. Certainly, our promising results make the use of quercetin a potential innovative approach, as well as an important methodological improvement for the preservation of other tissues.

## Data Availability

The data is contained within the article.
